# Supplementary material for: Mechanisms governing the pioneering and redistribution capabilities of the non-classical pioneer PU.1
Source: Nat Commun. 2020 Jan 21;11:402. doi: 10.1038/s41467-019-13960-2 (PMC6972792; doi:10.1038/s41467-019-13960-2)
Supplement: Supplementary file 7 — Source data [file 41467_2019_13960_MOESM7_ESM.zip › Source_Data/FigureS2/FigureS2B_MotifScanOutput/KmeansCluster6/homerResults/motif3.similar.html]

motif3

## Information for motif3

A
T
G
C
G
A
C
T
A
G
C
T
A
G
T
C
G
A
T
C
G
C
A
T
A
T
G
C
G
A
C
T
A
G
C
T
T
A
G
C
G
A
T
C
G
A
C
T
A
G
T
C
  
Reverse Opposite:  

T
C
A
G
C
T
G
A
C
T
A
G
A
T
C
G
T
C
G
A
C
T
G
A
T
A
C
G
C
G
T
A
C
T
A
G
T
C
A
G
T
C
G
A
C
T
G
A
T
A
C
G
  

|  |  |
| --- | --- |
| p-value: | 1e-310 |
| log p-value: | -7.148e+02 |
| Information Content per bp: | 1.724 |
| Number of Target Sequences with motif | 1651.0 |
| Percentage of Target Sequences with motif | 50.14% |
| Number of Background Sequences with motif | 8340.5 |
| Percentage of Background Sequences with motif | 19.39% |
| Average Position of motif in Targets | 100.4 +/- 47.4bp |
| Average Position of motif in Background | 99.2 +/- 88.7bp |
| Strand Bias (log2 ratio + to - strand density) | -0.1 |
| Multiplicity (# of sites on avg that occur together) | 1.30 |
| Motif File: | file (matrix) reverse opposite |

### Similar de novo motifs found

|  |  |  |  |  |  |  |  |
| --- | --- | --- | --- | --- | --- | --- | --- |
| Rank | Match Score | Redundant Motif | P-value | log P-value | % of Targets | % of Background | Motif file |
| 1 | 0.837 | G A C T G A T C A G C T A G T C A G T C G C A T A T G C A G C T A C G T A G T C A G T C A C G T A G T C G A T C | 1e-132 | -306.188719 | 17.13% | 4.80% | motif file (matrix) |
| 2 | 0.861 | T A C G C G T A C T A G A T C G C T G A C T A G T A C G C G T A C T A G C T A G C T G A T C A G | 1e-112 | -259.868839 | 13.18% | 3.34% | motif file (matrix) |
| 3 | 0.842 | A G C T A G T C A G T C G A C T A T G C A G T C G A C T G A T C G A T C A G C T G A T C | 1e-18 | -41.670138 | 2.34% | 0.64% | motif file (matrix) |
